# The COVID-19 Global Pandemic and Its Impact on the Mental Health of Nurses in Malaysia

**DOI:** 10.3390/healthcare9101259

**Published:** 2021-09-24

**Authors:** Ping Lei Chui, Mei Chan Chong, Khatijah Lim Abdullah, Vimala Ramoo, Li Yoong Tang, Wan Ling Lee, Chong Chin Che, Nor Aziyan Yahaya, Kavitha Rasaiah, Noor Hanita Zaini, Nor Zehan Ahmad, Chin Hai Teo

**Affiliations:** 1 Department of Nursing Science, Faculty of Medicine, Universiti Malaya, Kuala Lumpur 50603, Malaysia; mcchong@um.edu.my (M.C.C.); vimala@ummc.edu.my (V.R.); lytang@ummc.edu.my (L.Y.T.); wllee@g.ummc.edu.my (W.L.L.); chechongchin@um.edu.my (C.C.C.); aziyan@ummc.edu.my (N.A.Y.); lashia@um.edu.my (K.R.); noorhanita@um.edu.my (N.H.Z.); norzehan@um.edu.my (N.Z.A.); 2 Department of Nursing School of Healthcare, Sunway University, Petaling Jaya 47500, Malaysia; khatijahl@sunway.edu.my; 3 UM eHealth Unit, Dean’s Office, Faculty of Medicine, Universiti Malaya, Kuala Lumpur 50603, Malaysia; teoch@um.edu.my

**Keywords:** COVID-19 pandemic, coping strategy, depression, Malaysia, mental health, nurses, stress

## Abstract

The Coronavirus disease 2019 (COVID-19) global pandemic since its onset has had a dramatic and often devastating impact, both physical and psychological, on all healthcare workers. This study aimed to assess the impact of psychological distress that COVID-19 has on nurses, as well as the coping strategies that they employed. This is a cross-sectional national online survey. A total of 859 nurses actively involved in caring for patients with suspected or confirmed COVID-19 in Malaysia participated in the study. More than three-quarters of the nurses experienced stress (77.2%). A total of 88.7% and 7.2% of nurses revealed a moderate and high stress level, respectively. Approximately one in eight (12.1%) nurses reported feeling depressed. Nurses working in the outpatient departments reported significantly higher stress levels than nurses working in inpatient care departments. Nurses having chronic health problems reported significantly higher depression levels than nurses with no chronic health problem. Highly stressed or depressed nurses tend to adopt avoidance coping strategies while religion and emotional support were used regardless of the stress or depression levels experienced. The findings of the study provide insight into the mental health and coping strategies of nurses actively involved in caring for patients with suspected or confirmed COVID-19 in Malaysia. This would be of tremendous help to nursing administrators in implementing mental health services for nurses during and following the COVID-19 global pandemic.

## 1. Introduction

The Coronavirus disease 2019 (COVID-19) is an illness caused by severe acute respiratory syndrome coronavirus 2. COVID-19 is so highly contagious that by July 2021 it had infected more than 195 million people and caused more than 4 million deaths globally [1]. Such a high prevalence of an infectious viral disease would inevitably exert extraordinary pressure on any healthcare system and its workers. Healthcare workers are at risk of sacrificing their health and contracting the disease while performing their professional duties and even becoming a source of transmission in their community. The physical risks are further compounded by the concomitant risk of mental health issues [2]. It is a sad truth that more than 115,000 healthcare workers have lost their lives due to the coronavirus worldwide [1]. Nurses, who are at the frontline of providing life-saving care for COVID-19 patients, are placed at a significantly high risk of experiencing mass traumatization [3]. Their psychological stress is now recognized as a real problem [4,5]. Nurses suffer tremendous physical and psychological stress resulting from physical exhaustion from an increased workload, the risk of transmission, especially with an inadequate supply of personal protective equipment (PPE), and the ethical dilemma of triaging patient care [6]. A systematic review and meta-analysis found that the prevalence of depression (nurses: 30.30% vs. doctors: 25.37%) and anxiety (nurses 25.80% vs. doctors: 21.73%) was to be significantly higher among nurses than doctors during the COVID-19 pandemic [7]. Previous studies have shown that the prolonged wearing of protective gear, i.e., surgical masks, gloves, goggles, face shields, gowns, and N95 masks, has caused physical problems such as skin lesions, and the de novo PPE-associated headache [8]. In addition, the psychological distress experienced has included stress symptoms, such as reduced appetite or indigestion, fatigue, insomnia, nervousness, frequent crying, and even suicidal thoughts [9]. It is not surprising that junior and inexperienced nurses encounter greater stress levels. This is true of even experienced nurses dealing with a novel challenge. The psychological crisis can and will contribute to an adverse impact on the safety and quality of life of nurses [10].

In Malaysia, the onslaught of the COVID-19 pandemic was felt with the first wave of the outbreak that involved 22 people between 25 January to 15 February 2020 with no fatalities. The second wave of the outbreak was recorded as beginning on 27 February 2020. The number of people infected started to escalate and it reached 5959 by 29 April 2020 [11], despite the nation-wide movement control order (MCO) being implemented from 18 March until 14 April 2020. As of 29 July 2021, the country’s total number of coronavirus cases has reached 1,061,476 [11]. The Ministry of Health has mobilized nurses to work in designated COVID-19 hospitals with high numbers of confirmed COVID-19 cases. This group of nurses entrusted with the care of patients infected with COVID-19 is at the frontline risking their physical safety and mental health in upholding their professional oath of service. Self-care tips on physical and mental well-being for frontline personnel were issued by the authorities to help them cope with their workload. However, these proved inadequate given the lack of knowledge and information related to the virus, especially with regard to its severity and the treatments available, and the impact on healthcare workers, especially nurses, working in close proximity with COVID-19 patients. Stress is an occupational risk for all nurses, but especially significant for this group of nurses. Their level of risk for post-traumatic stress disorder (PTSD) had yet to be known, and the impact of demographic and cultural factors on their mental health had yet to be fully understood. Hence, it became absolutely necessary to examine their mental health and coping strategies during this period of uncertainty in order to develop early intervention programs and strategies to promote their physical and mental well-being [10]. To achieve this, the study aimed to assess the perceived stress, stress symptoms, and levels of depression experienced by nurses, their coping strategies, and the association of stress and depression with their demographic profiles given Malaysia’s unique ethnic pluralism.

## 2. Materials and Methods

### 2.1. Participants and Procedure

A cross-sectional online survey through a self-administered questionnaire was carried out during the implementation of movement control order (MCO) and conditional movement control order (CMCO) during the COVID-19 pandemic in Malaysia from April 2020 to August 2020. In order to obtain a sample population that best represents the entire population being studied, a stratified sampling method was used to divide the 27 hospitals designated for the treatment of patients with COVID-19 by the Ministry of Health Malaysia. The hospitals were classified under four strata based on their geographical location: viz. central, southern, and eastern regions of Peninsular Malaysia and East Malaysia. Three hospitals were selected from the central region (Hospital Sungai Buloh, Hospital Kuala Lumpur, and University Malaya Medical Centre), one from the southern region (Hospital Tunku Jaafar Seremban), one from the eastern region (Hospital Tengku Ampuan Afzan), and one from East Malaysia (Hospital Umum Sarawak) for a better representation of the study population. Three hospitals were chosen from the central region mainly because it has the highest number of cases of COVID-19. The target population for this study comprised nurses providing inpatient care and outpatient screening to patients with COVID-19. A non-probability convenient sampling technique was adopted. The inclusion criterion was nurses actively involved in caring for patients with suspected or confirmed COVID-19 in outpatient and inpatient facilities. The exclusion criteria were nursing students and nurses diagnosed with a pre-existing psychiatric illness.

### 2.2. Measures

The questionnaire consisted of four main parts. Part I comprised questions that solicited the demographic, social, and job-related characteristics of the nurses. Part II was the Perceived Stress Scale (PSS) that is used to measure perceived stress levels and elicit stress symptoms [12]. The PSS consisted of 10 items which evaluated the nurses’ perceived stress using a 5-point Likert scale (0 = never, 1 = almost never, 2 = sometimes, 3 = fairly often, and 4 = very often). The scores ranged from 0 to 40 and were categorized into three levels: low level (0–13), moderate level (14–26), and high level (27–40) [12]. The frequency of stress symptoms was assessed based on 12 common symptoms using the descriptors: frequent (almost all day/every day, once or twice daily, every night or day, 2–3 times per week, once a week) and not frequent (once a month or never). Part III utilized the Major Depression Inventory (MDI) (ICD-10) to measure the nurses’ level of depression [13]. The MDI (ICD-10) assessed the depression level using 10 items with a 5-point Likert scale: 0 (at no time), 1 (some of the time), 2 (less than half of the time), 3 (more than half of the time), 4 (most of the time), and 5 (all of the time). For items 8(a) versus 8(b) and items 10(a) versus 10(b), the highest score on (a) or (b) was used. The scores ranged from 0 to 50 and were categorized into four levels based on: no or doubtful depression (0–20); mild depression (21–25); moderate depression (26–30); and severe depression (31–50) [14]. Part IV consisted of the Brief COPE that determined the nurses’ coping strategies. The 28-item Brief-COPE scale comprised 14 dimensions. Each dimension consisted of two items rated on a 4-point Likert scale ranging from 1 (I haven’t been doing this at all) to 4 (have been doing this a lot) [15]. The scores ranged from 2 to 8. The 14 dimensions could be characterized primarily as either an avoidant coping strategy (self-distraction, substance use, behavioural disengagement, denial, venting, and self-blame) or an approach coping strategies (approach, emotional support, use of information support, positive reframing, planning, and acceptance). Humour and religion are neither avoidant coping nor approach coping strategies [15]. Adapted questionnaires are a well-established research tool within the health sciences. A minor modification was done after evaluation by the panel of experts on content validity. A pilot study was conducted on 30 nurses and the internal consistency of the instruments was found to be within the acceptable range. The Cronbach alpha coefficients of 0.70, 0.94, and 0.93 for the PSS, MDI, and Brief-COPE scale, respectively, indicated good reliability.

The online questionnaire link was emailed to the nursing officers in the six hospitals designated for the treatment of patients with COVID-19 and selected to participate in the study. The nursing officers then invited eligible nurses by sharing with them the email and questionnaire link. In the email, they were given clear and detailed explanations about the purpose, expected time required to complete the online questionnaire, confidentiality, and the right to decline participation or withdraw without giving any reason at any time. Data collection began after obtaining ethical approval from the University Malaya Medical Centre Medical Research Ethics Committee (MRECID.NO: 2020411-8502) and the Medical Research and Ethics Committee, Ministry of Health Malaysia (NMRR-20-890-54719-IIR). Participation was voluntary. Electronic consent was obtained from the participants by asking them to click on the ’agree’ button. Their responses were confidential as no name/email address/IP address was required. The study was performed in accordance with the ethical standards outlined in the 2008 Declaration of Helsinki and in compliance with the Strengthening the Reporting of Observational Studies in Epidemiology (STROBE) reporting guidelines.

### 2.3. Statistical Analyses

The data were analyzed using SPSS version 25.0 (IBM Corp., Armonk, NY, USA). Descriptive statistics were used to summarize and describe the demographic, social, and job-related characteristics of the nurses, their perceived level of stress and depression, stress symptoms, and coping strategies. Spearman’s correlation was used to measure the correlation between coping strategies and perceived levels of stress and depression. Multiple logistic regression was used to measure the association between demographic characteristics and stress/depression levels. A *p*-value of < 0.05 was interpreted as significant.

## 3. Results

### 3.1. Baseline Characteristics

A total of 1057 nurses responded to the online survey link, agreed, consented, and completed the study. However, 198 responses stated they did not take care of COVID-19 patients. Therefore, only 859 who provided outpatient and inpatient care for patients with suspected or confirmed COVID-19 were included in the analysis. The mean age of the participants was 32.7 (SD = 6.9) and their mean working experience was 9.3 years (SD = 6.5). The majority of them (94.6%, *n* = 813) had been working in hospital settings, with 85.9% (*n* = 738) employed as staff nurses. However, in terms of educational qualifications, most of them (89.2%, *n* = 766) had only a diploma in nursing. About two-thirds of them were married (67.6%, *n* = 581). Other demographic characteristics of the participants are displayed in Table 1.

### 3.2. Level of Stress and Depression

Table 2 shows the stress and depression levels among the participants of this study. The mean total score for stress was 21.2 (SD = 4.2). A total of 77.2% (*n* = 664) of the nurses experienced stress. A small percentage (7.2%, *n* = 62) indicated high-stress levels during the COVID-19 pandemic while the majority of them had moderate stress levels (88.7%, *n* = 762). As for depression, the mean total score was 12.4 (SD = 7.9). Out of the 12.1% (*n* = 104) of the participants who indicated signs of depression, their levels ranged from mild (5.8%, *n* = 50) to moderate (3.5%, *n* = 30) and severe depression (2.8%, *n* = 24).

The most common stress symptoms were fatigue (60.1%, *n* = 516), tense muscles, sore neck and back (47.7%, *n* = 410), eating too much or too little (42.1%, *n* = 362), and difficulty falling asleep (41.6%, *n* = 357), as evident in Table 3.

### 3.3. Correlation between Coping Strategies and Perceived Stress Scale; Coping Strategies and Major Depression Inventory

Religion (mean = 6.92, SD = 1.45) was the most commonly used coping strategy by the participants and this was followed by acceptance (mean = 6.07, SD = 1.61), positive reframing (mean = 6.02, SD = 1.68), active coping (mean = 5.93, SD = 1.60), and planning (mean = 5.81, SD = 1.59). The least favoured coping strategy was the use of emotional support. An approach coping strategy (total mean = 31.30, SD = 6.3) was more commonly used than an avoidant coping strategy (total mean = 26.5, SD = 6.89). Table 4 shows the detailed coping strategy employed by the participants during the COVID-19 pandemic.

In relation to stress, all the coping strategies except for religion and the use of emotional support showed a significant positive correlation with the PSS score. Three avoidant coping strategies: self-blame (r = 0.249), venting (r = 0.209), and substance use (r = 0.189) had the highest correlation coefficient among all the coping strategies, and the correlations were small [16]. As for depression, all the coping strategies, with the exception of religion, use of emotional support, and acceptance, had a significant positive correlation with the MDI score. Similar to perceived stress, self-blame (r = 0.485), venting (r = 0.405), and substance use (r = 0.393) had the highest correlation coefficient with MDI score among all the coping strategies, but these were medium correlations [16] as compared to stress. This shows that highly stressed or depressed participants tended to perform avoidance strategies while religion and emotional support were used regardless of stress or depression level (Table 4).

### 3.4. Association of Demographic Characteristics with the Perceived Level of Stress and Depression

In the multivariate logistic regression, factors with significance levels of less than 0.20 (*p* < 0.20) in the univariate analysis were included in the multivariate analysis. The multiple logistics regression (Table 5) shows that nurses working in the outpatient department reported a significantly higher stress level (AOR 3.740; 95% CI 1.983–7.053; *p* < 0.001) while nurses having chronic health problems reported a significantly higher depression level (AOR 4.687; 95% CI 2.268–9.682; *p* < 0.001).

## 4. Discussion

The primary finding of this nationwide study is that more than three-quarters of the nurses experienced stress during the COVID-19 pandemic. This finding is consistent with studies performed in China [17,18,19]. This implies that stress was particularly significant in the early stages of the COVID-19 outbreak as the data in this study were collected during the second wave of the COVID-19 outbreak in Malaysia. The lack of proper guidelines and ineffective communication in the early/first stage of the COVID-19 pandemic were the most likely stressors among the nurses, especially with rapidly changing information and emerging facts about the contagion [20,21]. Nurses rely on their organizations to provide them with reliable information and knowledge about the pandemic that is easy to comprehend and is delivered in a consistent manner [22]. If this is available, it would definitely alleviate their stress. Stress among healthcare workers can and does impair their attention, cognitive functioning, and decision-making in the clinical area [23], while overwhelming stress can lead to burnout, thus jeopardizing patient safety [24]. The findings of the study indicate that immediate action is required to avoid and/or ameliorate/mitigate potential mental health problems.

The present study found that most of the nurses experienced a variety of physical and/or psychological manifestations of stress while managing patients during the COVID-19 pandemic. The most common symptoms were fatigue, tense muscles, sore neck and back pain, overeating or loss of appetite, and insomnia. Our findings are mostly consistent with other published works which state that healthcare workers, particularly nurses, who come in close contact with COVID-19 patients when providing care, often encounter problems such as fear of being infected due to inadequate working conditions, such as a lack of adequate infection control practices, anxiety, and depression [25,26,27]. Previous studies have also revealed that when nurses provide direct care to patients with infectious diseases, such as SARS, Ebola, 9 MERS-CoV, and H1N1, they suffer anxiety, loneliness, fatigue, sleep disorders, and other mental and physical health problems [28,29,30]. Their anxiety and fears are often linked to seeing their colleagues being infected and this is further aggravated by the instances of death among their peers not only due to COVID-19, but also other ailments or cardiac arrest caused by overwork and fatigue. Therefore, in a pandemic, early psychological interventions and perpetual screening and assessment of stress levels among nurses are imperative. The goal should be to provide professional, flexible, and continuous psychological intervention to promote the physical and emotional health of nurses.

The present study found that approximately one in eight (12.1%) of the nurses were suffering from mild to severe depression during the COVID-19 pandemic. This is higher than a study conducted in 40 government-designated hospitals in China that revealed that 9.4% of the nurses suffered from depressive symptoms [31]. Likewise, a study performed in two major tertiary institutions in Singapore reported that 8.9% of health care workers screened positive for depression [32]. However, this is considerably lower when compared with a systematic review and meta-analysis, which found that 22.8% of healthcare workers suffered from depression during the COVID-19 pandemic [7]. Since some of the studies did not single out nurses, it is evident from the current study that there is a high prevalence of depression among nurses handling COVID-19 patients. Early identification and immediate action are required to prevent the situation from worsening given that the global pandemic shows no sign of abating in the new future.

The top three coping strategies adopted by nurses to overcome stress during the COVID-19 pandemic were discovered to be religion, acceptance, and positive reframing. These coping strategies are emotion-focused and tend to outweigh other coping strategies when nurses perceive that a stressor is something that they must undertake or cannot avoid [33]. A study conducted in China reported that nurses are inclined to adopt problem-focused strategies in coping with stress during the COVID-19 pandemic [34]. The present researchers/authors are of the opinion that Malaysian nurses are prepared to fulfill their duty in the pandemic situation, believing that they have a professional obligation to provide their patients with standard care under any circumstances. In addition, Malaysians live in a multicultural society and that has inculcated in them an emotional tolerance towards others irrespective of race/ethnicity or religion. Thus, they are more prone to employ emotion-focused coping strategies. Turning to religion to cope with stress yielded the highest score among the coping strategies employed by Malaysian nurses. Religion can serve as a basis of emotional support, as a method for positive reinterpretation and growth, thereby helping the individual to deal with /manage a variety of personal and collective stressors [35]. Surprisingly, seeking emotional support from third parties was the least popular psychological stress-coping strategy among nurses. In this study, the nurses were inclined to seek information rather than emotional support from others. A possible explanation could be that hospital policies and practice guidelines were being revised rapidly and consistently in the early pandemic stage. It is believed that nurses perceive constructive advice and accurate information to be far more important than moral support at this critical period. The present study found that highly stressed or depressed participants tend to adopt avoidant coping strategies such as self-blame, venting, and substance use. Unfortunately, this type of passive coping strategy that is centered on emotional distress is significantly correlated to poor psychological health, including post-traumatic stress, anxiety, and depression symptoms [36].

The study indicates that nurses working in the outpatient department experienced significantly higher stress levels than those dealing with inpatient care. A possible explanation could be that outpatient nurses are the gatekeepers responsible for screening suspected cases and handling the confirmed cases of COVID-19 before admission into the ward. The decision-making process can be a stressor. The current study also found that nurses with existing chronic health problems experienced a significantly higher depression level than fit and healthy nurses. This could be because the former category of nurses is postulated to be at a higher risk of contracting COVID-19 but are nevertheless obligated to be part of the frontline for combating the virus [37].

This is a national online study that assessed the impact of the COVID-10 pandemic on the mental health of nurses in Malaysia. The findings of this study are generalizable to the whole country as the data were collected from a sizable sample of 859 nurses serving in six of the COVID-19 hospitals designated by the Malaysian Ministry of Health in various regions of Peninsular and East Malaysia. However, these results must be interpreted with caution. These are several limitations to the study. It was conducted during the initial stage of the outbreak, and it focused on the short-term psychological effects on nurses only. In terms of study design, a longitudinal study is recommended since it is a better tool to assess the long-term effects of the pandemic on nurses’ psychological health. A qualitative study that encourages nurses to share their lived experience in caring for patients during the COVID-19 pandemic is highly recommended as it would allow researchers to explore the specific factors contributing to their stress and their personal coping strategies.

## 5. Conclusions

Nurses’ mental health is an essential element in occupational health and safety under any circumstances. It is even more imperative to establish better support for nurses to cope with stress during a pandemic such as the present COVID-19 crisis. The findings of the study provide insight into the mental wellbeing and coping strategies of nurses actively involved in caring for patients with suspected or confirmed COVID-19 in Malaysia. This would be of tremendous help in enlightening nursing administrators in implementing mental health services for nurses both during and following the global COVID-19 global pandemic.

## Figures and Tables

**Table 1:** Characteristics of nurses (N = 859).

Characteristic		*n* (%)
Age (Mean, SD)	32.7 (6.9) years	-
Working experience as a nurse (Mean, SD)	9.3 (6.5) years	-
Current work setting		
Hospital		813 (94.6)
Clinic/Community Health Centre		46 (5.4)
Type of patient care		
Inpatient		765 (89.1)
Outpatient		94 (10.9)
Designation		
Nurse manager		95 (11.1)
Staff nurses		738 (85.9)
Assistance nurses		26 (3.0)
Nursing educational level		
Certificate		33 (3.8)
Diploma		766 (89.2)
Degree/Master/PhD		60 (7.0)
Have any chronic health problem		
Yes		53 (6.2)
No		808 (93.8)
Marital status		
Married		581 (67.7)
Single/widow/divorced		278 (32.3)
Number of children		
0		377 (43.9)
1–2		294 (34.2)
3–4		169 (19.7)
≥5		19 (2.2)

**Table 2:** Perceived Stress Scale and Major Depression Inventory score of the participants (N = 859).

Variable	*n* (%)
Perceived Stress Scale	Mean = 21.2, SD = 4.2
Low (0–13)	35 (4.1)
Moderate (14–26)	762 (88.7)
High (27–40)	62 (7.2)
Major Depression Inventory	Mean = 12.4, SD = 7.9
No (0–20)	755 (87.9)
Mild (21–25)	50 (5.8)
Moderate (26–30)	30 (3.5)
Severe (31–50)	24 (2.8)

Note: SD = Standard Deviation.

**Table 3:** Stress Symptoms (N = 859).

Symptoms	Frequent *n* (%)
Fatigue	516 (60.1)
Tense muscles, sore neck and back	410 (47.7)
Eating too much or too little	362 (42.1)
Difficulty falling asleep	357 (41.6)
Headaches	339 (39.5)
Irritability	308 (35.9)
Insomnia	271 (31.5)
Anxiety, worry, phobias	269 (31.3)
Bouts of anger/hostility	240 (27.9)
Diarrhea, cramps, gas, constipation	193 (22.5)
Boredom, depression	190 (22.1)
Restlessness, itching	155 (18.0)

Note: Frequent = at least once a week.

**Table 4:** COPE score of the participants and its correlation with Perceived Stress Scale (PSS) and Major Depression Inventory (MDI) score.

Coping Strategy	Mean	SD	Spearman’s Rho vs. PSS	Spearman’s Rho vs. MDI
Self-distraction (avoidant)	5.73	1.52	0.166 **	0.277 **
Active coping (approach)	5.93	1.60	0.131 **	0.130 **
Substance use (avoidant)	3.84	1.57	0.189 **	0.393 **
Emotional support (approach)	2.16	0.72	0.067	0.049
Use of information support (approach)	5.31	1.72	0.086 *	0.167 **
Behavioural disengagement (avoidant)	5.41	1.73	0.110 **	0.149 **
Denial (avoidant)	3.17	1.44	0.176 **	0.298 **
Venting (avoidant)	4.69	1.59	0.209 **	0.405 **
Positive reframing (approach)	6.02	1.68	0.133 **	0.108 **
Planning (approach)	5.81	1.59	0.149 **	0.154 **
Humour	4.43	1.81	0.119 **	0.221 **
Acceptance (approach)	6.07	1.61	0.121 **	0.050
Religion	6.92	1.45	0.065	0.030
Self-blame (avoidant)	3.67	1.55	0.249 **	0.485 **
Sum of all avoidants	26.50	6.30	0.269 **	0.493 **
Sum of all approaches	31.30	6.89	0.150 **	0.143 **

Note: ** *p* < 0.01; * *p* < 0.05. SD = Standard Deviation.

**Table 5:** Univariate and multivariate logistic regression on perceived stress and depression (N = 859).

Characteristics	Stress (PSS)				Depression (MDI)			
	OR (95% CI)	*p*-Value	AOR (95% CI)	*p*-Value	OR (95% CI)	*p*-Value	AOR (95% CI)	*p*-Value
Age	0.978 (0.939–1.018)	0.269	-	-	0.933 (0.899–0.968)	<0.001	1.008 (0.917–1.109)	0.867
Working experience as nurse (year)	0.967 (0.925–1.011)	0.137	0.972 (0.915–1.032)	0.350	0.920 (0.884–0.958)	<0.001	0.909 (0.821–1.005)	0.063
Current work setting		0.445	-	-		0.242		0.550
Hospital	1.753 (0.415–7.408)				2.033 (0.619–6.675)		1.470 (0.416–5.192)	
Clinic/Community Health Centre	1				1		1	
Type of patient care								
Inpatient	1		1		1			
Outpatient	3.206 (1.733–5.930)	<0.001	3.740 (1.983–7.053)	<0.001	1.312 (0.714–2.413)	0.381	-	-
Designation								
Nurse manager	1		1		1		1	
Staff nurses	1.393 (0.542–3.576)	0.491	0.992 (0.300–3.276)	0.150	1.857 (0.835–4.131)	0.129	0.646 (0.230–1.820)	0.409
Assistance nurses	3.273 (0.811–13.21)	0.096	3.027 (0.670–13.67)	0.055	1.048 (0.204–5.374)	0.956	0.534 (0.095–3.016)	0.477
Nursing educational level			-	-			-	-
Certificate	1.517 (0.378–6.089)	0.557			0.900 (0.210–3.860)	0.887		
Diploma	0.818 (0.314–2.129)	0.680			1.274 (0.534–3.043)	0.585		
Degree/Master/PhD	1				1			
Have any chronic health problem			-	-				
Yes	1.369 (0.524–3.573)	0.521			2.856 (1.493–5.464)	0.002	4.687 (2.268–9.682)	<0.001
No	1				1		1	
Marital status								
Married	1		1		1		1	-
Single/widow/divorced	1.678 (0.994–2.833)	0.053	1.687 (0.936–3.038)	0.082	2.140 (1.413–3.241)	<0.001	1.492 (0.872–2.554)	0.145
Number of children	0.943 (0.784–1.134)	0.530	-	-	0.740 (0.624–0.878)	0.001	0.960 (0.757–1.218)	0.738

Note: For PSS, the outcomes were grouped into low/moderate vs. high; For MDI, the outcomes were grouped into no depression vs. low/moderate/high depression.

## Data Availability

Data available on request from the authors.
